# circRNA-ZCCHC14 affects the chondrogenic differentiation ability of peripheral blood-derived mesenchymal stem cells by regulating GREM1 through miR-181a

**DOI:** 10.1038/s41598-023-29561-5

**Published:** 2023-02-18

**Authors:** Daohong Zhao, Hong Chen, Jia Zhong, Xizong Zhou, Jun Zhang, Yuhao Zhang

**Affiliations:** 1 Department of Orthopaedics, The Second Affiliated Hospital of Kunming Medical University, Kunming, China; 2 Department of Sports Medicine, The First People’s Hospital of Kunming City, Kunming, China; 3 Department of Orthopaedics, The People’s Hospital of XiShuangBanNa State, Jinghong, China; 4 Department of Orthopaedics, The People’s Hospital of YanJing County, Zhaotong, China

**Keywords:** Cell biology, Stem cells

## Abstract

circRNAs play an important role in the progression of osteoarthritis (OA). Therefore, we aimed to reveal the mechanism of action of circRNA-ZCCHC14 in OA. OA animal and cell models were constructed, and clinical samples were collected. The expression of circRNA-ZCCHC14 and miR-181a was detected by RT‒qPCR. The chondrogenic differentiation ability of peripheral blood-derived mesenchymal stem cells (PBMSCs) was detected by Alcian blue staining. The expression of chondrogenic differentiation-related proteins was detected by Western blotting. Double fluorescein experiments verified the targeting relationship of miR-181a with circRNA-ZCCHC14 and GREM1. Upregulation of circRNA-ZCCHC14 was observed in blood, in BMP-2- and TGF-β3-treated PBMSCs from OA patients and in animal models. Knockdown of circRNA-ZCCHC14 promoted the chondrogenic differentiation ability of PBMSCs. circRNA-ZCCHC14 was found to bind to miR-181a and negatively regulate miR-181a expression. Inhibition of miR-181a reversed the promoting effect of circRNA-ZCCHC14 knockdown on the chondrogenic differentiation ability of PBMSCs. GREM1 was identified as a target of miR-181a. Overexpression and knockdown of GREM1 regulated the expression of BMP2, which in turn affected the chondrogenic differentiation ability of PBMSCs, indicating that GREM1 and BMP2 have antagonistic effects and that they jointly regulate the chondrogenic differentiation of PBMSCs. circRNA-ZCCHC14 may promote the chondrogenic differentiation ability of PBMSCs by regulating miR-181a and inhibiting the expression of GREM1.

## Introduction

Osteoarthritis (OA) is a common chronic joint disease characterized by changes in osteochondral homeostasis leading to progressive degeneration of synovial joints^1^. OA can lead to joint pain, deformity, and dysfunction, thereby reducing the patient’s quality of life. Some studies have shown that the incidence of OA in the middle-aged population is 40%, while the incidence rate in the elderly is 80% or higher. Mild OA can lead to joint pain, deformity, and functional impairment affecting quality of life, while more severe cases can lead to disability^2^. The disability rate can eventually reach 50% or more, placing a heavy economic burden on society^3^. Current clinical treatment methods, such as conservative drug treatment, joint debridement, grinding, microfracture, and autologous cartilage transplantation, have limited therapeutic effects^4–6^. Therefore, new strategies need to be developed to treat OA.

Circular RNA (circRNA) is an endogenous noncoding RNA (ncRNA) with a covalently closed loop structure, neither a 3' tail nor a 5' cap^7^. This structural property enables it to exhibit high stability, high evolutionary conservation among species, and tissue specificity in the eukaryotic transcriptome^8^. Several studies have shown that circRNAs play a key role in the pathogenesis of various orthopaedic diseases (osteoporosis, intervertebral disc degeneration, osteoarthritis, etc.)^9,10^. However, the roles of circRNAs and the circRNA and miRNA axes in OA development and progression are poorly understood. In this study, we explored the role of circRNA-ACCHC14 in OA progression.

MicroRNAs (miRNAs) are a group of small noncoding single-stranded RNAs approximately 17–24 nucleotides in length. In recent years, studies have confirmed that miRNAs play an important regulatory role in the differentiation of stem cells into chondrocytes^11^. It has been found that miR-146a is involved in the regulation of TGF-β signalling during chondrocyte development^12^. Inhibition of LEF-1 by miR-449a repressed the expression of SOX9, resulting in a slowing of cartilage formation^13^. Studies have shown that miR-140, a miRNA related to chondrocyte differentiation, is downregulated in OA cartilage and that changes in miR-140 expression and function play an important role in diseases affecting articular cartilage^14^. miR-181a is involved in the process of osteogenic differentiation and is highly expressed during osteogenic differentiation^15^. In addition, miR-181a promotes osteogenic differentiation by downregulating the expression of TGF-β^16^. The above phenomenon suggests that miR-181a is closely related to stem cell differentiation. Our previous study found that miR-181a was upregulated in PBMSCs treated with BMP-2 and TGF-β3 and was positively correlated with chondrogenesis-related markers^17^.

GREM1 is a highly conserved glycoprotein, a member of the DAN Cerberus family^18^, and mainly distributed in the extracellular matrix, but a small amount of GREM1 is also distributed in the endoplasmic reticulum^19^. Studies have shown that GREM1 is an inhibitor of the process by which bone morphogenetic proteins regulate osteogenic differentiation^20^. GREM1 binds to BMP-2, -4 and -7 and inhibits their binding to BMP receptors on the cell membrane^21^. In osteoblasts, overexpression of GREM1 reduces the biological activity of BMP-2, and reducing GREM1 expression in osteoblasts using RNA interference increases the biological activity of BMP-2^21^.

Although many circRNAs have been reported to play critical roles in OA, the specific microenvironmental factors involved in this pathological process have not been fully elucidated. Therefore, studying the effect of circRNAs on OA progression may help to elucidate the pathogenesis of OA. Our study revealed that si-circRNA-ACCHC14 inhibits the expression of GREM1 by promoting miR-181a, which may provide a new diagnostic and therapeutic strategy for OA.

## Materials and methods

### Patient samples and ethics statement

Peripheral blood samples from 10 OA patients and 7 healthy people were collected from the Second Affiliated Hospital of Kunming Medical University. The characteristics of the subjects enrolled in the OA study are shown in Table 1. The diagnosis of patients with OA was based on the American College of Rheumatology guidelines. This research scheme follows the ethical principles of the Helsinki Declaration and was approved by the Ethics Committee of Clinical Research of the Second Affiliated Hospital of Kunming Medical University (Shen-PJ-Ke-2022-64). All the selected patients signed an informed consent form.

**Table 1:** Characteristics of the subjects enrolled in the study of osteoarthritis.

Characteristic	Healthy donor	Patients
Case, n	7	10
Age, (range)	40.50 (28–52)	64.65 (54–75)
Sex, n (%)		
Male	4 (57.1)	4 (40)
Female	3 (42.9)	6 (60)

### Animal model

All surgical procedures and protocols were carried out in accordance with the Guide to Nursing and Use of Experimental Animals and approved by the Ethics Review Committee of Animal Experiments of Kunming Medical University (kmmu20221858). All animal methods are reported in accordance with ARRIVE guidelines.

The establishment of animal models was performed according to previous literature^22^. Full thickness defects were created in adult Yunnan Xiaoer pigs (n = 6, male or female, average weight 15 kg). Briefly, pentobarbital sodium was injected into the ear vein for anaesthesia induction. The right joint was cut through a medial approach at the patellar tendon, and then, the patella was dislocated in an external position, exposing the joint cavity. A full thickness defect was created through the surface of the medial and lateral femoral cartilage with a drill (drilling: 7 mm in diameter, 4 mm in depth). After successful modelling, the Diannan Xiaoer pigs were kept in a controlled environment with free access to food and water. All animals were studied simultaneously at the same age (the average age was 9 months). At 30 days after surgery, blood (30 ml) was collected from the porcine anterior vena cava in a 5 ml vacuum collection tube containing heparin sodium. In addition, a 20 ml sterile syringe was used to puncture the medial side of the patellar ligament of the knee into the joint cavity at 45 degrees to collect the joint fluid.

### Isolation and culture of PBMSCs

PBMSCs were isolated and cultured according to a previous study^17^. Briefly, the peripheral blood of Xiaoer pigs was collected, diluted with D-Hanks solution, and subjected to Ficoll density gradient centrifugation to directly separate and purify peripheral blood mononuclear cells. Mononuclear cells were collected and cultured in serum-free medium and placed in an incubator at 37 °C with 5% CO_2_.

### Cell transfection

The PBMSCs (80%-90% confluency) were digested with 0.25% trypsin containing EDTA and inoculated into six-well plates at a cell density of 2 × 10^4^ cells/cm^2^. The differentiated PBMSCs were transfected with si-circRNA-ACCHC14, oe-circRNA-ACCHC143, si-GREM1, oe-GREM1, si-BMP2, oe-BMP2, miR-181a mimic, miR-181a inhibitor or their negative controls using Lipofectamine 2000 transfection reagent (Invitrogen, Carlsbad, CA, USA). After transfection, the cells were cultured in an incubator at 37 °C with 5% CO_2_ for 48 h.

### RT‒qPCR

Total RNA was extracted from peripheral blood using TRIzol reagent (Invitrogen, Carlsbad, CA, USA). The concentration was then measured using an ultratrace UV analyser. RNA was reverse transcribed into cDNA according to the Bestar qPCR RT Kit instructions. PCR amplification was then performed with DBI Bestar^®^ SYBR Green qPCR Master Mix (DBI Bioscience, Shanghai, China) using a QuantStudio 6 Flex Real-Time PCR System (Applied Biosystems). The expression levels of miRNAs and circRNAs were normalized against U6 or GAPDH expression, and relative quantification was performed using the 2^−ΔΔCt^ method. Primers are shown in Table 2.

**Table 2:** Primer sequences.

Gene	Sequence(5’-3’)
GAPDH	F:TGTTCGTCATGGGTGTGAAC
	R:ATGGCATGGACTGTGGTCAT
CircRNA-ZCCHC14	F:TTTGCGGTCATCAGACTTCCT
	R:TTGCCACAGCATTCTGAAACA
U6	F:CTCGCTTCGGCAGCACA
	R:AACGCTTCACGAATTTGCGT
miR-181a	F:CTCAACTGGTGTCGTGGAGTCGGCAATTCAGTTGAGACTCACCG
	R:ACACTCCAGCTGGGAACATTCAACGCTGTCG

### Alcian blue staining

PBMSCs were seeded on 24-well plates and induced using chondrogenic differentiation medium for 14 days. Cells were then fixed with 4% paraformaldehyde and stained with Alcian blue dye solution (Solarbio, China). Finally, the cells were photographed with an inverted optical microscope (Leica DMI 3000B, Germany).

### Western blot analysis

Total protein was extracted using RIPA buffer (Beyotime Biotechnology), and its concentration was quantified using a BCA protein assay kit (Thermo). Equal amounts of protein were subjected to 10% SDS‒PAGE and transferred to PVDF membranes (Millipore, USA). Membranes were blocked with 5% bovine serum albumin (BSA) (Amresco, USA) and incubated with specific primary antibodies (anti-GAPDH (Abcam), anti-BMP2 (Abcam), anti-COL2A1 (Abcam), and anti-AGR (Abcam), followed by incubation with HRP-conjugated secondary immunoglobulin antibodies (Boster). After enhanced chemiluminescence (ECL) colour development, gel imager images were acquired. Finally, the protein bands were quantitatively analysed with ImageJ software.

### Statistical analysis

Data tables were analysed using GraphPad Prism 6.0 (GraphPad, USA). For a normal distribution, a t test was used to assess differences between two groups, while one-way analysis of variance (ANOVA) was used for comparisons among three or more groups. P < 0.05 was considered statistically significant.

## Results

### Expression of circRNA-ZCCHC14 and miR-181a

**Figure 1:**
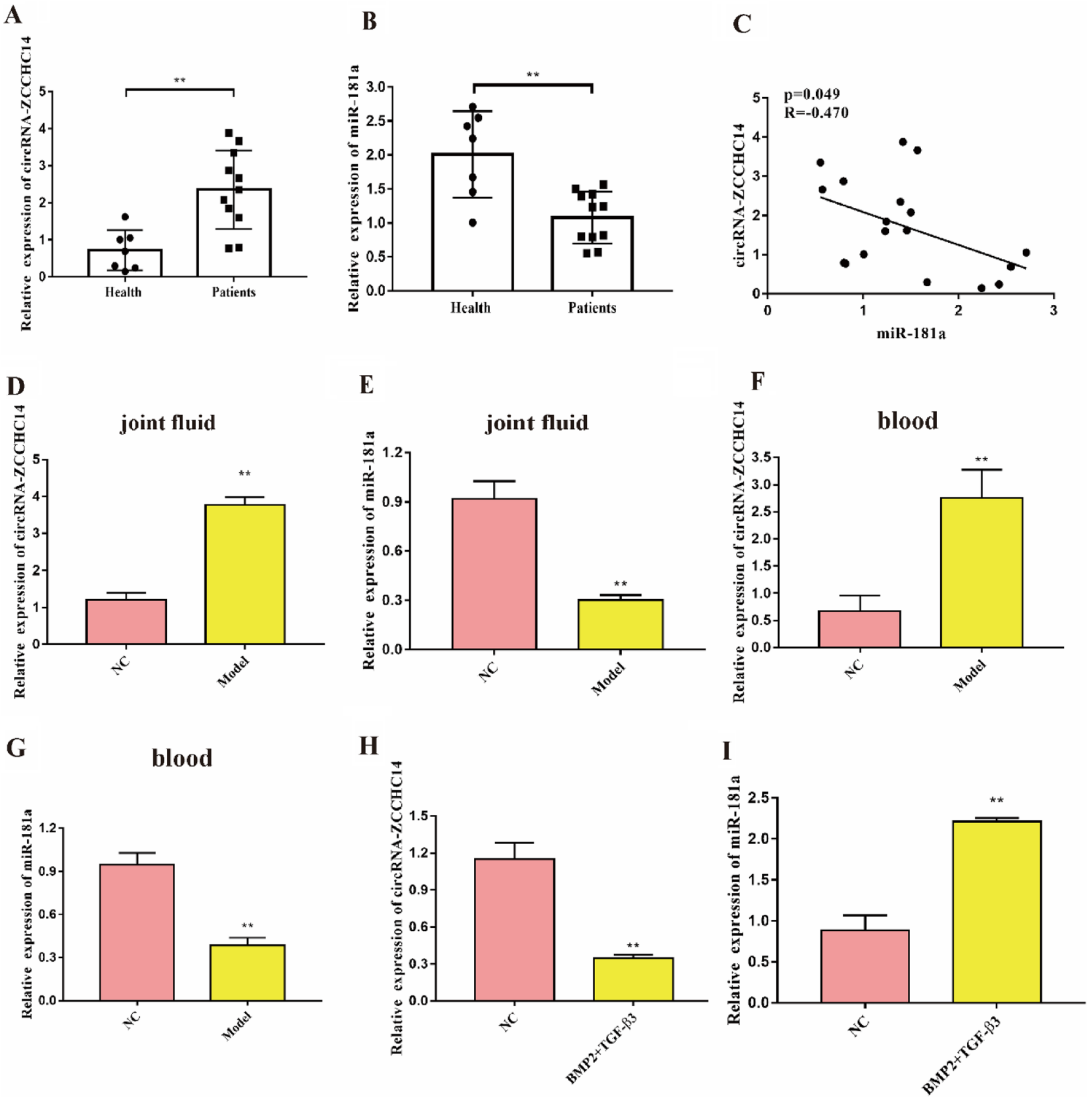
Expression of circRNA-ZCCHC14 and miR-181a (**A**) Detection of the circRNA-ZCCHC14 expression in clinical samples by RT‒qPCR; (**B**) Detection of miR-181a expression in clinical samples by RT‒qPCR; (**C**) The correlation between circRNA-ZCCHC14 and miR-181a was analysed; (**D**) RT‒qPCR was used to detect the expression of circRNA-ZCCHC14 in animal joint fluid; (**E**) RT‒qPCR was used to detect the expression of miR-181a in animal joint fluid; (**F**) The expression of circRNA-ZCCHC14 in animal blood was detected by RT‒qPCR; (**G**) RT‒qPCR was used to detect the expression of miR-181a in animal blood; (**H**) Detection of the expression of circRNA-ZCCHC14 in the cell model by RT‒qPCR; (**I**) Detection of the expression of miR-181a in the cell model by RT‒qPCR.

We examined the expression of circRNA-ZCCHC14 and miR-181a at three levels: cellular, clinical and animal. Clinical samples were collected to detect the expression of circRNA-ZCCHC14 and miR-181a, and it was found that miR-181a expression was significantly lower in the blood of patients, while circRNA-ZCCHC14 was highly expressed (Fig. 1A,B). Pearson correlation analysis revealed that circRNA-ZCCHC14 was negatively correlated with miR-181a (Fig. 1C). An OA animal model was constructed, and blood and joint fluid were collected to detect the expression of circRNA-ZCCHC14 and miR-181a (Fig. 1D–G). Using a combination of BMP2 and TGF-β to induce cells, it was found that the expression of circRNA-ZCCHC14 was decreased, while the expression of miR-181a was increased (Fig. 1H,I). Taken together, miR-181a was negatively correlated with circRNA-ZCCHC14, which was associated with chondrogenic differentiation.

### The effect of circRNA-ZCCHC14 on the chondrogenic differentiation of PBMSCs

**Figure 2:**
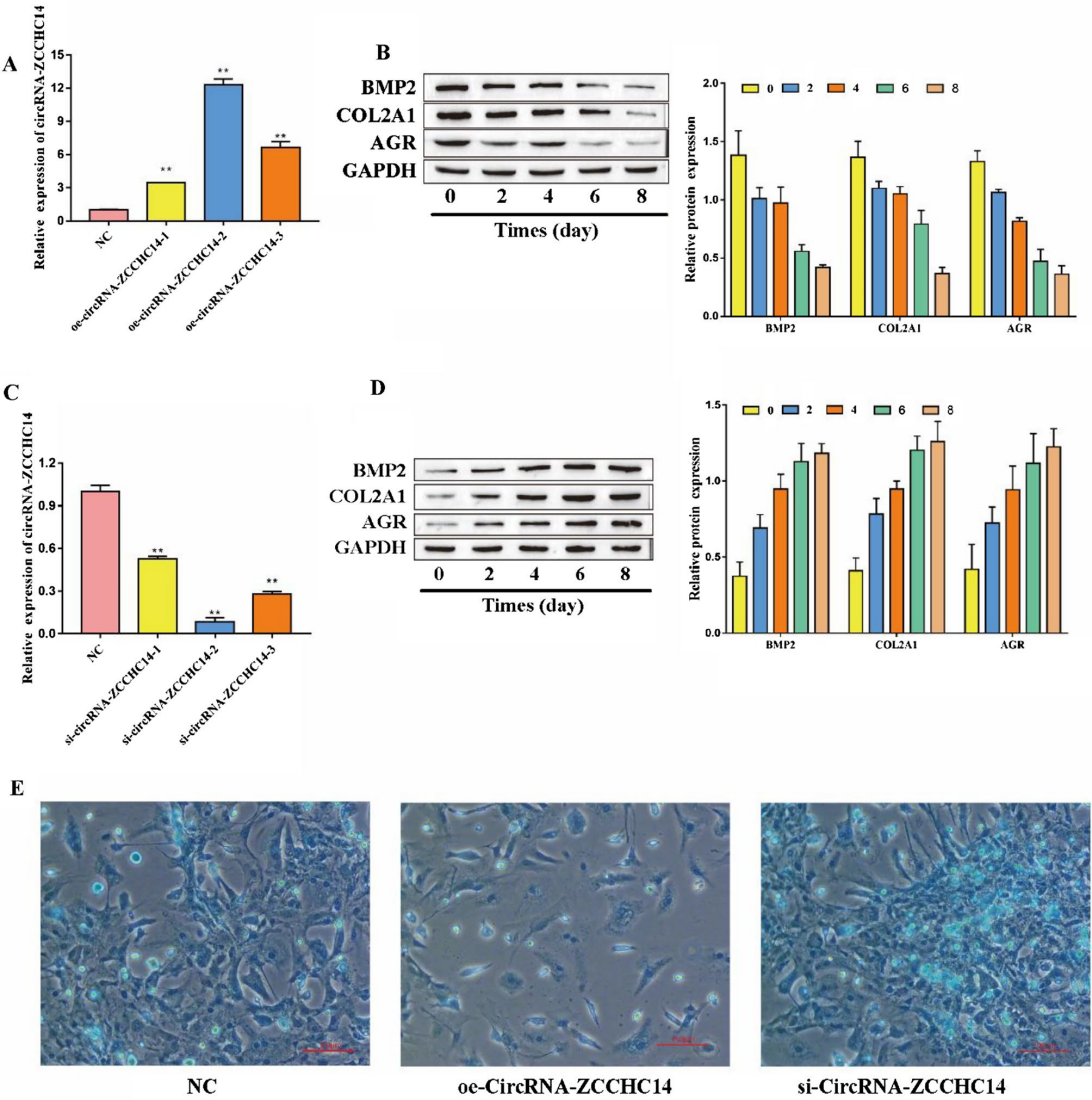
The effect of circRNA-ZCCHC14 on the chondrogenic differentiation of PBMSCs (**A**) RT‒qPCR detection of circRNA-ZCCHC14 overexpression efficiency; (**B**) Western blot detection of the expression of the cartilage markers BMP2, COL2A1 and AGR; (**C**) RT‒qPCR detection of the circRNA-ZCCHC14 knockdown efficiency; (**D**) Western blotting was used to detect the expression of the cartilage-forming markers BMP-2, COL-2A1 and AGR; (**E**) Alcian blue staining was used to detect the ability of cells to form cartilage.

To explore the effect of circRNA-ZCCHC14 on the chondrogenic differentiation of PBMSCs, we detected the circRNA-ZCCHC14 knockdown or overexpression efficiencies via RT‒qPCR. The results showed that the overexpression vector significantly increased the expression of circRNA-ZCCHC14 in all three experiments, and the overexpression effect of oe-circRNA-ZCCHC14-2 was the best (Fig. 2A). Therefore, oe-circRNA-ZCCHC14-2 was selected for subsequent experiments (hereafter, oe-circRNA-ZCCHC14 refers to oe-circRNA-ZCCHC14-2). The chondrogenesis markers BMP2, COL2A1 and AGR were detected by Western blot, and it was found that the chondrogenic markers decreased with increasing treatment time with oe-circRNA-ZCCHC14-2 (Fig. 2B). In addition, all siRNAs significantly reduced circRNA-ZCCHC14 expression, with si-circRNA-ZCCHC14-2 having the highest knockdown efficiency (Fig. 2C). Therefore, the si-circRNA-ZCCHC14-2 interfering cell line was used for subsequent experiments (hereafter, si-circRNA-ZCCHC14 refers to si-circRNA-ZCCHC14-2). The chondrogenesis markers BMP-2, COL-2A1 and AGR were detected by Western blot, and it was found that the chondrogenic markers increased with increasing treatment time with si-circRNA-ZCCHC14-2 (Fig. 2D). The ability of cells to form cartilage was detected by Alcian blue staining. The results showed that the ability of the oe-circRNA-ZCCHC14 group to form cartilage was decreased compared with that of the NC group, while the ability of the si-circRNA-ZCCHC14 group to form cartilage was increased (Fig. 2E). In conclusion, circRNA-ZCCHC14 inhibits the chondrogenic differentiation of PBMSCs.

### circRNA-ZCCHC14 targets and regulates miR-181a

**Figure 3:**
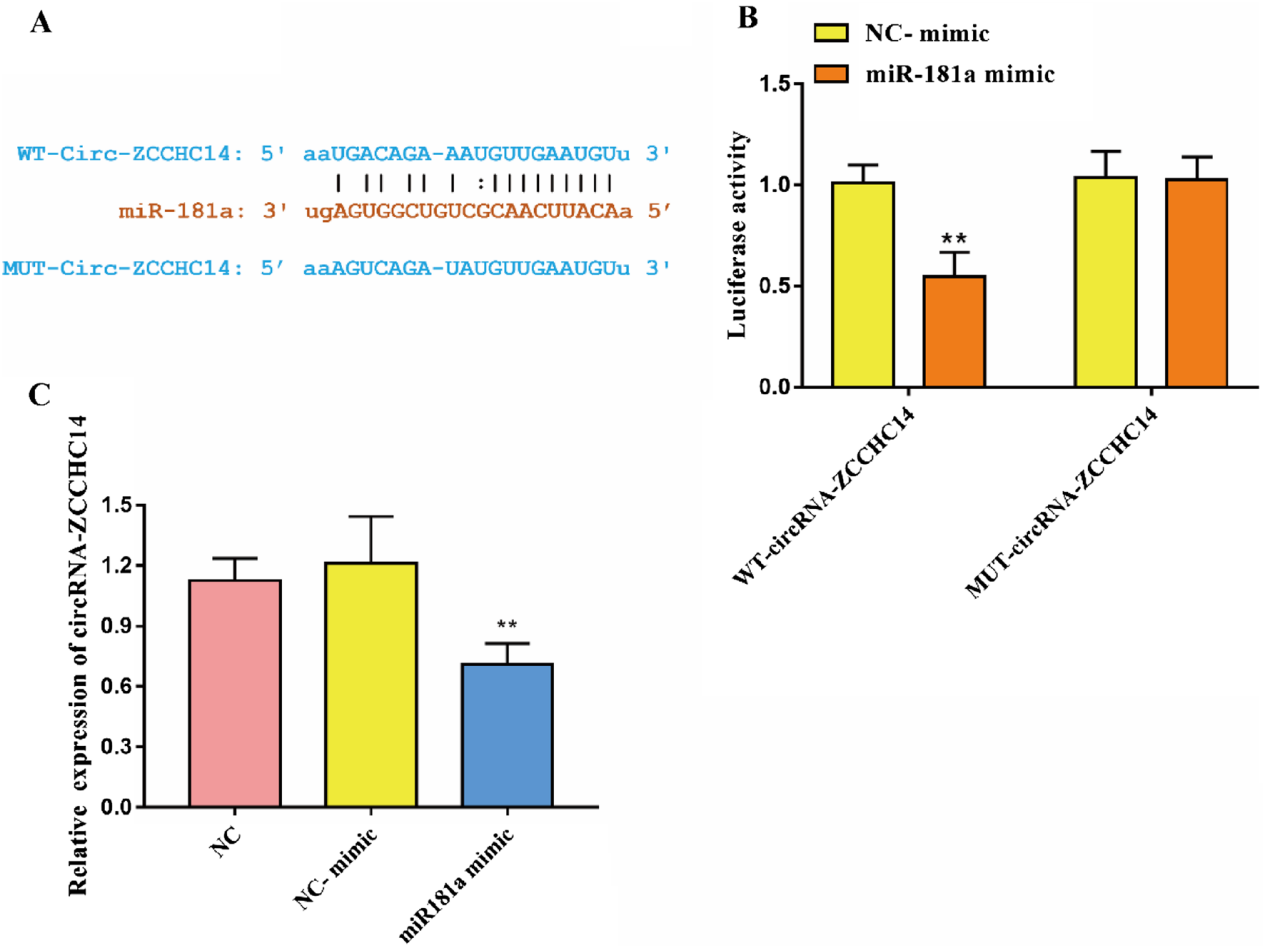
circRNA-ZCCHC14 targets miR-181a (**A**) circRNA-ZCCHC14 and miR-181a binding sequence; (**B**) Dual-luciferase reporter system to verify the binding of circRNA-ZCCHC14 and miR-181a; (**C**) RT‒qPCR detection of the expression level of circRNA-ZCCHC14.

The targeted binding sites between circRNA-ZCCHC14 and miR-181a were predicted using bioinformatics tools (Fig. 3A). Then, the sequence of circRNA-ZCCHC14 was mutated. After that, the targeting relationship between them was verified with a dual-luciferase assay, and it was found that the fluorescence intensity in the WT-circRNA-ZCCHC14 + miR-181a mimic group was significantly reduced, but the MUT-circRNA-ZCCHC14 + miR-181a mimic had no significant effect on fluorescence intensity (Fig. 3B). The expression of circRNA-ZCCHC14 was detected by RT‒qPCR, and it was found that miR-181a mimic significantly inhibited the expression level of miR-181a (Fig. 3C). These results suggest that circRNA-ZCCHC14 targets and regulates circRNA-ZCCHC14.

### circRNA-ZCCHC14 affects the chondrogenic differentiation of PBMSCs through miR-181a

**Figure 4:**
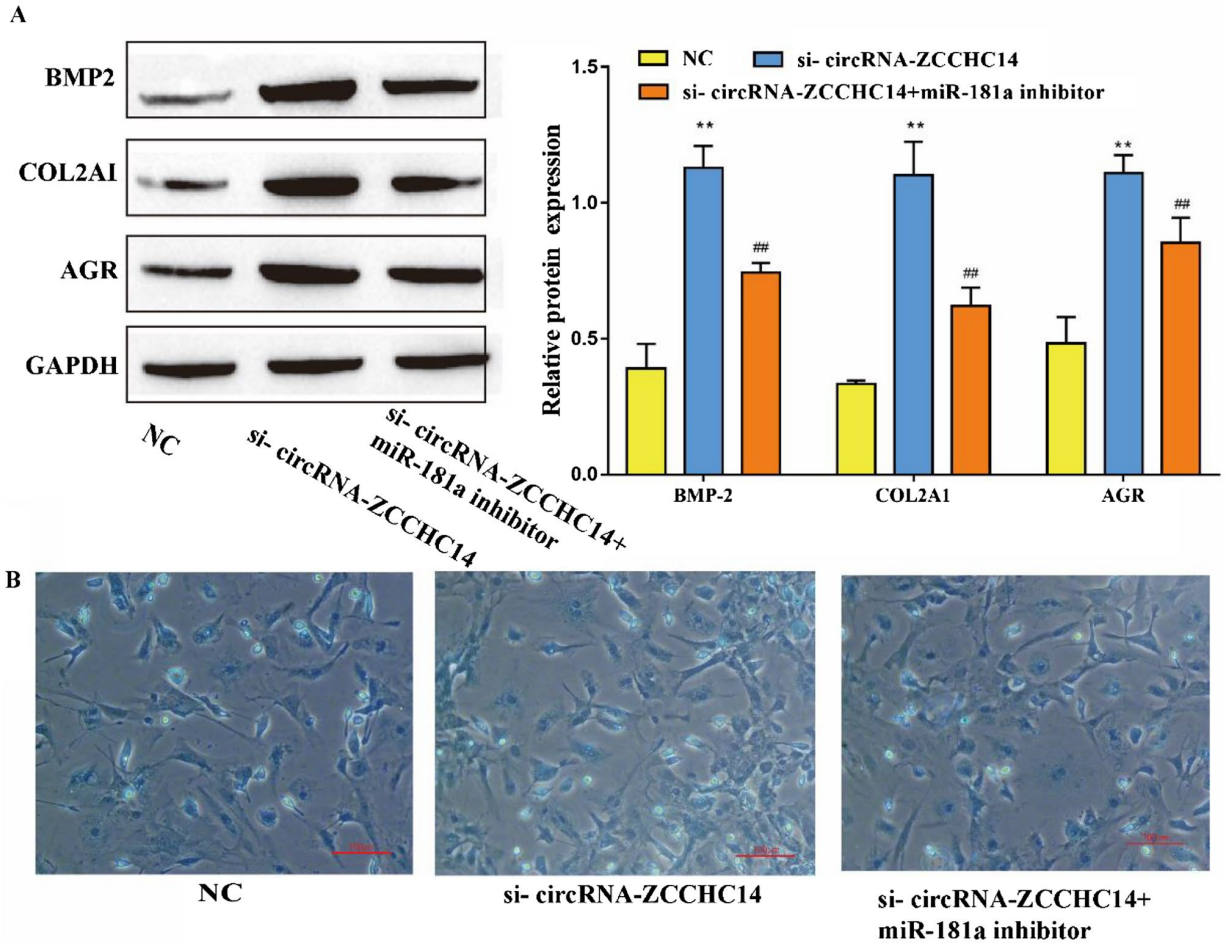
circRNA-ZCCHC14 affects PBMSC chondrogenic differentiation through miR-181a (**A**) Western blot detection of the cartilage markers BMP2, COL2A1 and AGR; (**B**) Alcian blue staining to detect the ability of cells to form cartilage.

After verifying the targeting relationship between circRNA-ZCCHC14 and miR-181a, we further explored whether circRNA-ZCCHC14 affects PBMSC chondrogenic differentiation through miR-181a. Compared with the NC group, si-circRNA-ZCCHC14 promoted the expression of the chondrogenesis markers BMP2, COL2A1 and AGR, while the miR-181a inhibitor significantly reversed this promotion (Fig. 4A). Using Alcian blue staining to detect the ability of cells to form cartilage, the same conclusion was reached; that is, si-circRNA-ZCCHC14 promoted the ability of cells to form cartilage, whereas the miR-181a inhibitor reversed this promotion (Fig. 4B). In conclusion, circRNA-ZCCHC14 affects the chondrogenic differentiation of PBMSCs through miR-181a.

### Validation of the targeting relationship between GREM1 and miR-181a

**Figure 5:**
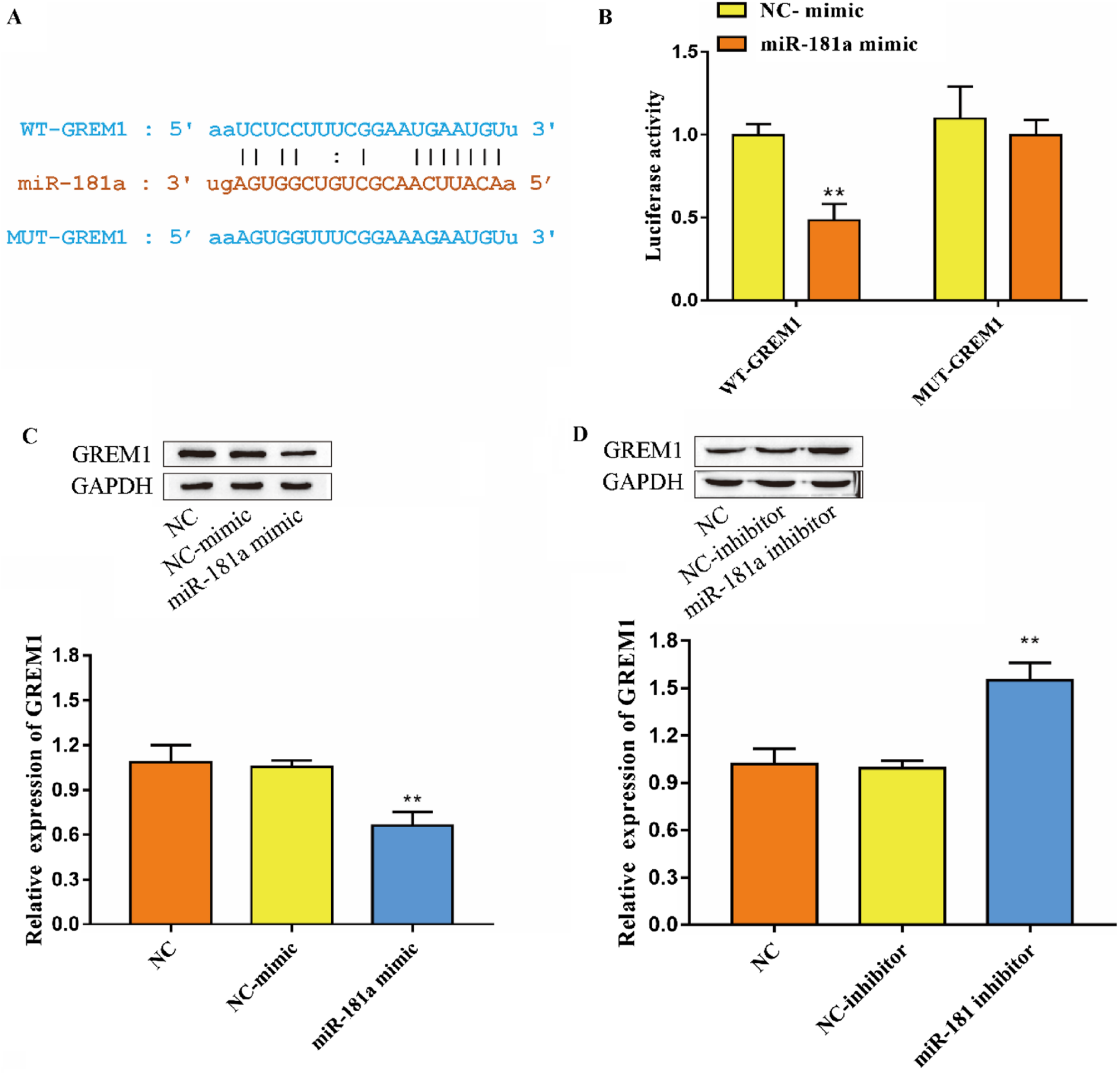
Validation of the targeting relationship between GREM1 and miR-181a (**A**) GREM1 and miR-181a binding sequence; (**B**) Dual-luciferase reporter system to verify the binding of GREM1 and miR-181a sequence; (**C**) Western blot detection of the expression level of GREM1; (**D**) Western blot detection of the expression level of GREM1.

The targeting relationship between GREM1 and miR-181a was predicted using starBase, and it was found that there was a target binding site between GREM1 and miR-181a (Fig. 5A). The targeting relationship was verified by a double fluorescein experiment, and it was found that the fluorescence activity in the WT-GREM1 + miR-181a mimic group was significantly reduced, but the MUT-GREM1 + miR-181a mimic had no significant effect on the fluorescence activity (Fig. 5B). The expression of GREM1 was detected by Western blotting, and it was found that miR-181a significantly inhibited the expression level of GREM1 (Fig. 5C,D). The above results indicate that there is a targeted negative regulatory relationship between miR-181a and GREM1.

### The effects of GREM1 and BMP2 on chondrogenic differentiation of PBMSCs

**Figure 6:**
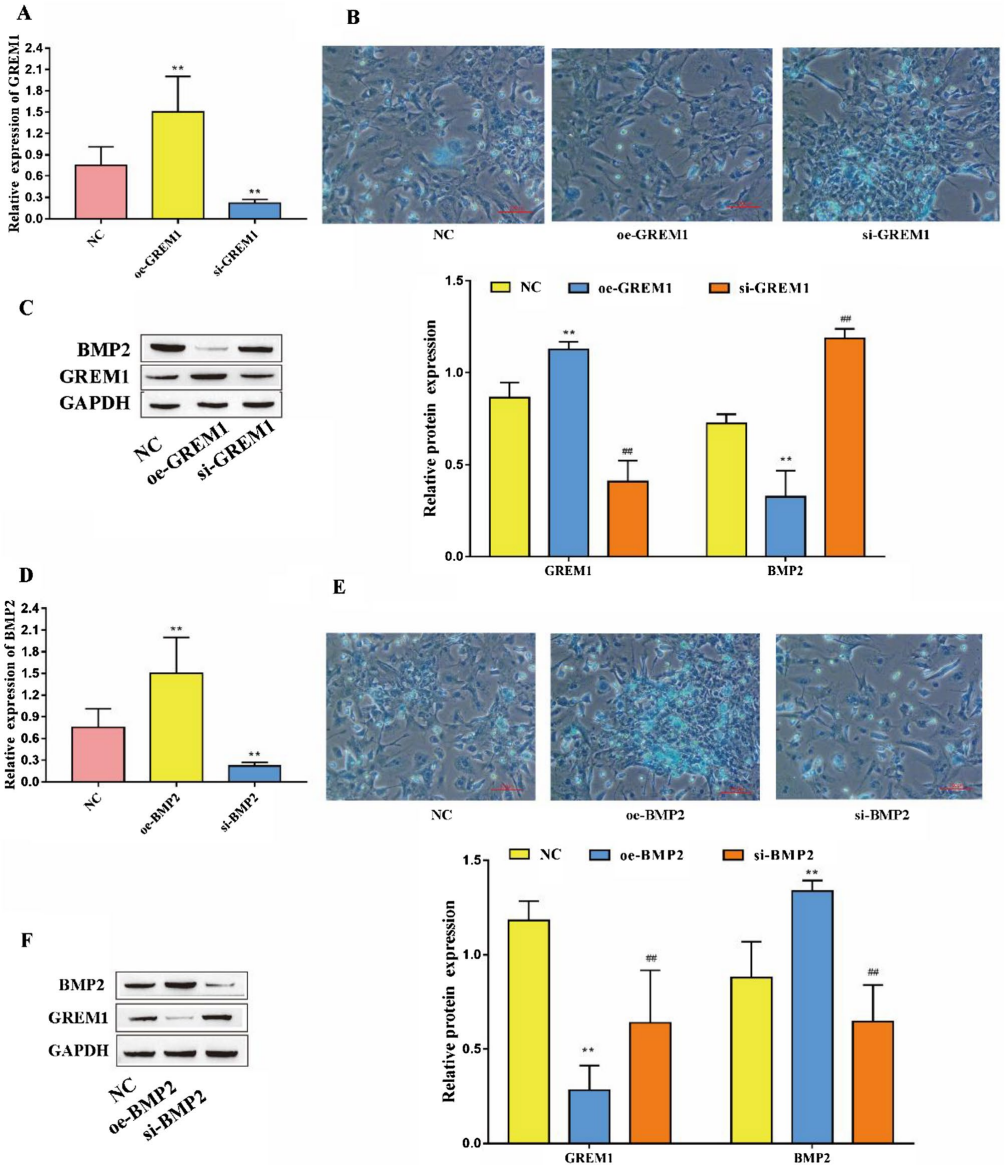
Effects of GREM1 and BMP2 on the chondrogenic differentiation of PBMSCs (**A**) RT‒qPCR analysis to detect the expression of GREM1; (**B**) Alcian blue staining to detect the ability of cells to form cartilage; (**C**) Western blotting to detect the expression of BMP-2 and GREM1; (**D**) RT‒qPCR analysis to detect the expression of BMP2; (**E**) Alcian blue staining was used to detect the ability of cells to form cartilage; (**F**) Western blotting was used to detect the expression of BMP-2 and GREM1.

We successfully constructed GREM1-overexpressing and GREM1-knockdown PBMSC lines (Fig. 6A). Alcian blue staining showed that overexpression of GREM1 reduced PBMSC chondrogenic differentiation and that knockdown of GREM1 increased PBMSC chondrogenic differentiation (Fig. 6B). Western blot analysis showed that overexpression of GREM1 reduced BMP2 expression; conversely, knockdown of GREM1 increased BMP2 expression (Fig. 6C). At the same time, we constructed BMP2 overexpression and knockdown PBMSC lines (Fig. 6D). Using Alcian blue staining, it was found that overexpression of BMP2 increased the chondrogenic differentiation of PBMSCs and that knockdown of BMP2 decreased the chondrogenic differentiation of PBMSCs (Fig. 6E). Western blot analysis showed that overexpression of BMP2 reduced GREM1 expression; conversely, knockdown of BMP2 increased GREM1 expression (Fig. 6F). The above results indicate that GREM1 and BMP2 have antagonistic effects and that they jointly regulate the chondrogenic differentiation of PBMSCs.

## Discussion

OA is one of the most common diseases that causes joint pain and affects joint function^3,23^. To date, there is no effective recommend OA treatment method in clinical settings. Therefore, new and in-depth research on the pathogenesis of OA is an important way to find new and effective therapies for the treatment of OA. BMP-2 and TGF-β3 belong to the transforming growth factor β superfamily and have the highest efficiency in inducing chondrogenic differentiation of BMSCs^24^. As a chondrogenic differentiation factor, BMP-2 mainly plays a role in chondrogenic differentiation in the early stage, while TGF-β3 plays an important role throughout the entire process, maintaining the cartilage phenotype, regulating the proliferation of chondrocytes, and promoting the production of collagen and proteoglycans. The two act at different stages of chondrogenic differentiation, and their combined application should play a synergistic role in inducing and regulating the directional differentiation of PBMSCs into chondrocytes^25^. How circRNAs promote the chondrogenic differentiation capacity of PBMSCs induced by BMP-2 and TGF-β3 has thus far remained elusive. In the present study, decreased expression of circRNA-ZCCHC14 and increased expression of miR-181a were found in clinical OA peripheral blood samples and in animal OA blood and joint fluid samples, and it was found that the expression levels of the two were negatively correlated in the peripheral blood of patients with OA.

circRNAs are widely present in human cells^26^. Compared with other noncoding RNAs, circRNAs are more stable and conserved and have the potential to be used as new diagnostic molecular biomarkers and therapeutic targets^27,28^. Multiple studies have found that circRNAs play an important role in OA. For example, circ_0000423 regulates cartilage ECM synthesis through the miRNA-27b-3p/MMP-13 axis^29^. TGF-β1 regulates chondrocyte proliferation and extracellular matrix synthesis in osteoarthritis through the circPhf21a-Vegfa axis^30^. circCDK14 ameliorated IL-1β-induced osteoarthritis chondrocyte injury through the miR-1183/KLF5 pathway^31^. The present study found that knockdown of circRNA-ZCCHC14 enhanced the chondrogenic differentiation ability of PBMSCs. In addition, starBase combined with dual-luciferase experiments verified that there is a targeted negative regulatory relationship between circRNA-ZCCHC14 and miR-181a.

miRNAs have important functions in stemness maintenance and differentiation of stem cells. Several studies have found that miR-181a is associated with osteogenic differentiation^32^. miR-181a is involved in the osteogenic differentiation process and is highly expressed during osteogenic differentiation^33^. miR-181a promotes osteogenic differentiation by downregulating the expression of TGF-β. miR-181a plays a negative regulatory role in cartilage homeostasis by inhibiting Ccna2 and Acan^34^. Our previous study found that miR-181a was significantly upregulated in PBMSCs treated with BMP-2 and TGF-β3.

To further determine the functional mechanism of circRNA-ZCCHC14, the downstream target genes of miR-181a were investigated. GREM1 is a highly conserved glycoprotein that has been widely demonstrated to be involved in organogenesis and tissue differentiation. Sequencing analysis revealed increased expression of GREM1 in OA samples, and high expression of GREM1 was associated with severe knee OA^35,36^. In addition, GREM1 was found to be highly expressed in IL-1β-induced chondrocytes, and its overexpression attenuated the functional effects of miR-183-5p, thereby promoting extracellular matrix degradation and chondrocyte apoptosis^37^. In the present study, we found that miR-181a can target and regulate GREM1 to affect the chondrogenic differentiation ability of PBMSCs, that GREM1 and BMP2 have antagonistic effects, and that they jointly regulate the chondrogenic differentiation of PBMSCs (Supplementary Figure).

## Conclusions

Our current study suggests that circRNA-ZCCHC14 is strongly expressed in OA patients, in an animal model and in PBMSCs treated with BMP-2 and TGF-β3. Knockdown of circRNA-ZCCHC14 inhibited the antagonistic effect of GREM1 on BMP2 by promoting the expression of miR-181a, thereby greatly improving the chondrogenic differentiation ability of PBMSCs. This study is the first to propose that miR-181a/GREM1 signalling is targeted by circRNA-ZCCHC14, enriching the evidence indicating that circRNA-ZCCHC14 is involved in OA progression. Promoting the chondrogenic differentiation ability of PBMSCs by inhibiting circRNA-ZCCHC14 may be an important strategy for future OA treatment.

### Mechanism diagram

**Figure 7:**
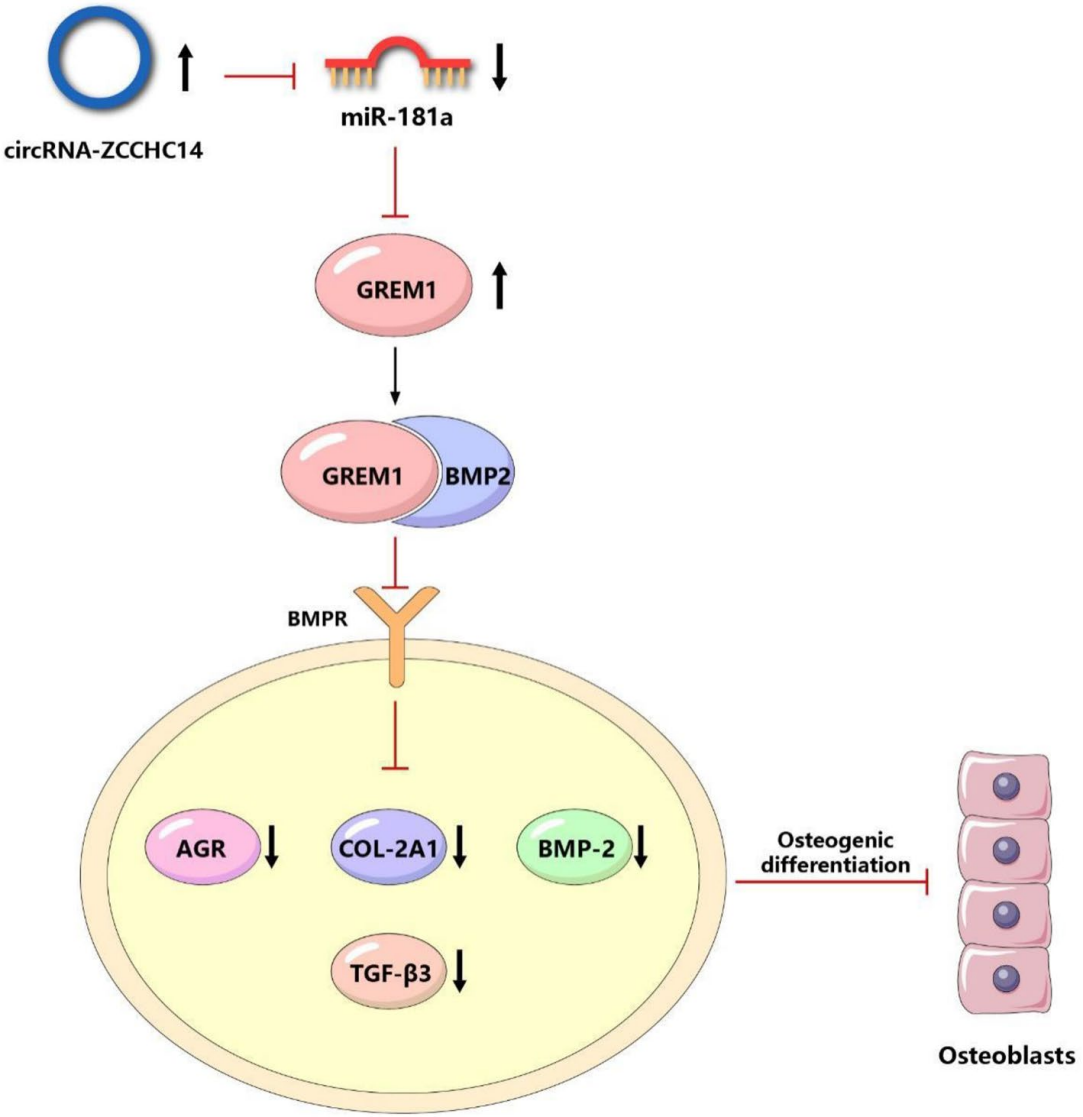
Mechanism diagram of how circRNA-ZCCHC14 affects the chondrogenic differentiation ability of peripheral blood mesenchymal stem cells by regulating GREM1 through miR-181a.

The mechanism diagram shows (Fig. 7) that the highly expressed circRNA-ZCCHC14 can indirectly promote the expression of GREM1 by inhibiting miR-181a, resulting in a decrease in the expression of chondrogenesis markers (BMP-2, COL-2A1, AGR, TGF-β3), and ultimately inhibiting chondrogenic differentiation.

## Supplementary Information

Supplementary Figures.

## Data Availability

All data supporting the findings of this study are contained within the article.

## References

[1] Pereira D, Peleteiro B, Araujo J (2011). The effect of osteoarthritis definition on prevalence and incidence estimates: a systematic review. Osteoarthr. Cartil..

[2] Song Y, Zhang J, Xu H (2020). Mesenchymal stem cells in knee osteoarthritis treatment: A systematic review and meta-analysis. J. Orthop. Translat..

[3] Glyn-jones S, Palmer AJ, Agricola R (2015). Osteoarthritis. Lancet.

[4] Kraeutler MJ, Belk JW, Purcell JM (2018). Microfracture versus autologous chondrocyte implantation for articular cartilage lesions in the knee: A systematic review of 5-year outcomes. Am. J. Sports Med..

[5] Nguyen VT, Cancedda R, Descalzi F (2018). Platelet lysate activates quiescent cell proliferation and reprogramming in human articular cartilage: Involvement of hypoxia inducible factor 1. J. Tissue Eng. Regen. Med..

[6] Worthen J, Waterman BR, Davidson PA (2012). Limitations and sources of bias in clinical knee cartilage research. Arthroscopy.

[7] Salzman J, Gawad C, Wang PL (2012). Circular RNAs are the predominant transcript isoform from hundreds of human genes in diverse cell types. PLoS ONE.

[8] Hentze MW, Preiss T (2013). Circular RNAs: Splicing’s enigma variations. Embo J..

[9] Zhi F, Ding Y, Wang R (2021). Exosomal hsa_circ_0006859 is a potential biomarker for postmenopausal osteoporosis and enhances adipogenic versus osteogenic differentiation in human bone marrow mesenchymal stem cells by sponging miR-431-5p. Stem Cell Res. Ther..

[10] Mao X, Cao Y, Guo Z (2021). Biological roles and therapeutic potential of circular RNAs in osteoarthritis. Mol. Ther. Nucleic Acids.

[11] Shang J, Liu H, Zhou Y (2013). Roles of microRNAs in prenatal chondrogenesis, postnatal chondrogenesis and cartilage-related diseases. J. Cell. Mol. Med..

[12] Cheung KS, Sposito N, Stumpf PS (2014). MicroRNA-146a regulates human foetal femur derived skeletal stem cell differentiation by down-regulating SMAD2 and SMAD3. PLoS ONE.

[13] Paik S, Jung HS, Lee S (2012). miR-449a regulates the chondrogenesis of human mesenchymal stem cells through direct targeting of lymphoid enhancer-binding factor-1. Stem Cells Dev..

[14] Miyaki S, Nakasa T, Otsuki S (2009). MicroRNA-140 is expressed in differentiated human articular chondrocytes and modulates interleukin-1 responses. Arthritis Rheum..

[15] Zhai X, Meng R, Li H (2017). miR-181a modulates chondrocyte apoptosis by targeting glycerol-3-phosphate dehydrogenase 1-like protein (GPD1L) in osteoarthritis. Med. Sci. Monit..

[16] Bhushan R, Grunhagen J, Becker J (2013). miR-181a promotes osteoblastic differentiation through repression of TGF-β signaling molecules. Int. J. Biochem. Cell Biol..

[17] Zhao D, Li Y, Li Y (2018). MiR-181a regulates the chondrogenic differentiation in pig peripheral blood mesenchymal stem cells. Int. J. Clin. Exp. Pathol..

[18] Huang X, Post JN, Zhong L (2018). Dickkopf-related protein 1 and gremlin 1 show different response than frizzled-related protein in human synovial fluid following knee injury and in patients with osteoarthritis. Osteoarthr. Cartil..

[19] Yin M, Tissari M, Tamminen J (2017). Gremlin-1 is a key regulator of the invasive cell phenotype in mesothelioma. Oncotarget.

[20] Hu K, Sun H, Gui B (2017). Gremlin-1 suppression increases BMP-2-induced osteogenesis of human mesenchymal stem cells. Mol. Med. Rep..

[21] Tomlinson IP, Carvajal-carmona LG, Dobbins SE (2011). Multiple common susceptibility variants near BMP pathway loci GREM1, BMP4, and BMP2 explain part of the missing heritability of colorectal cancer. PLoS Genet..

[22] Jia D, Zhang R, He Y, Cai G, Zheng J, Yang Y, Li Y (2021). Comparative effectiveness of two methods for inducing osteoarthritis in a novel animal model, the Diannan small-ear pig. J. Orthop. Surg. Res..

[23] Vinatiee C, Merceron C, Guicheux J (2016). Osteoarthritis: From pathogenic mechanisms and recent clinical developments to novel prospective therapeutic options. Drug Discov. Today.

[24] Rui Y, Du L, Wang Y (2010). Bone morphogenetic protein 2 promotes transforming growth factor β3-induced chondrogenesis of human osteoarthritic synovium-derived stem cells. Chin. Med. J..

[25] Wang X, Li Y, Han R (2014). Demineralized bone matrix combined bone marrow mesenchymal stem cells, bone morphogenetic protein-2 and transforming growth factor-β3 gene promoted pig cartilage defect repair. PLoS One.

[26] Jeck WR, Sorrentino JA, Wang K (2013). Circular RNAs are abundant, conserved, and associated with ALU repeats. RNA.

[27] Li F, Li C, Li X (2020). Altered circular RNA expression profiles in the non-ischemic thalamus in focal cortical infarction mice. Aging (Albany NY).

[28] Li P, Chen S, Chen H (2015). Using circular RNA as a novel type of biomarker in the screening of gastric cancer. Clin. Chim. Acta..

[29] Li X, Xie C, Xiao F (2022). Circular RNA circ_0000423 regulates cartilage ECM synthesis via circ_0000423/miRNA-27b-3p/MMP-13 axis in osteoarthritis. Aging (Albany NY).

[30] Lin S, Li H, Wu B (2022). TGF-β1 regulates chondrocyte proliferation and extracellular matrix synthesis via circPhf21a-Vegfa axis in osteoarthritis. Cell Commun. Signal..

[31] Lai X, Song Y, Tian JJA (2022). CircCDK14 ameliorates interleukin-1β-induced chondrocyte damage by the miR-1183/KLF5 pathway in osteoarthritis. Autoimmunity.

[32] Bhushan R, Grunhagen J, Becker J (2013). miR-181a promotes osteoblastic differentiation through repression of TGF-β signaling molecules. Int. J. Biochem. Cell Biol..

[33] Sumiysshi K, Kubota S, Ohgawara T (2013). Novel role of miR-181a in cartilage metabolism. J. Cell. Biochem..

[34] Ohgawara T, Kubota S, Kawaki H (2009). Regulation of chondrocytic phenotype by micro RNA 18a: Involvement of Ccn2/Ctgf as a major target gene. FEBS Lett..

[35] Yi J, Jin Q, Zhang B (2016). Gremlin-1 concentrations are correlated with the severity of knee osteoarthritis. Med. Sci. Monit..

[36] Fu M, Huang G, Zhang Z (2015). Expression profile of long noncoding RNAs in cartilage from knee osteoarthritis patients. Osteoarthr. Cartil..

[37] Jiang R, Gao H, Cong F (2021). Circ_DHRS3 positively regulates GREM1 expression by competitively targeting miR-183-5p to modulate IL-1β-administered chondrocyte proliferation, apoptosis and ECM degradation. Int. Immunopharmacol..

