# Supplementary material for: circRNA-ZCCHC14 affects the chondrogenic differentiation ability of peripheral blood-derived mesenchymal stem cells by regulating GREM1 through miR-181a
Source: Sci Rep. 2023 Feb 18;13:2889. doi: 10.1038/s41598-023-29561-5 (PMC9938902; doi:10.1038/s41598-023-29561-5)
Supplement: Supplementary file 1 — Supplementary Figures. [file 41598_2023_29561_MOESM1_ESM.docx]

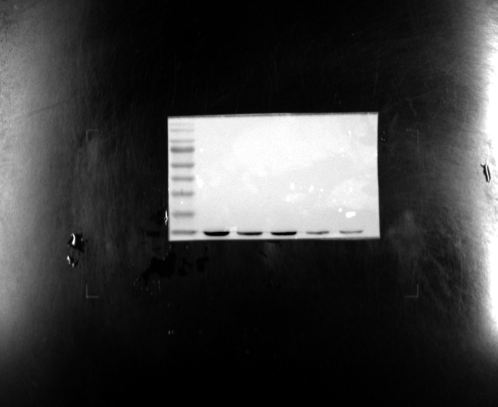

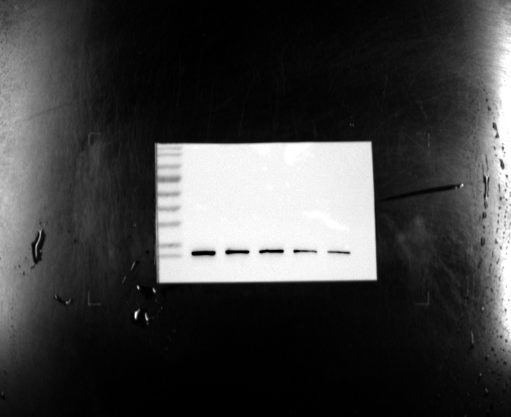


AGR.tif BMP2.tif


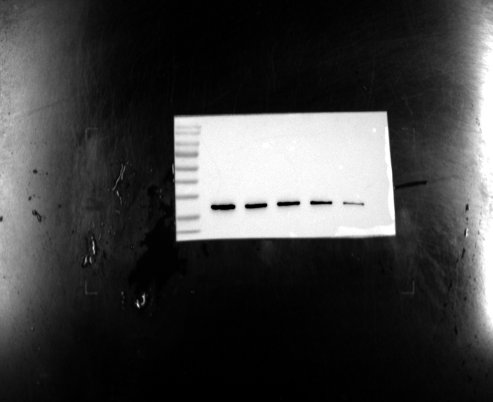

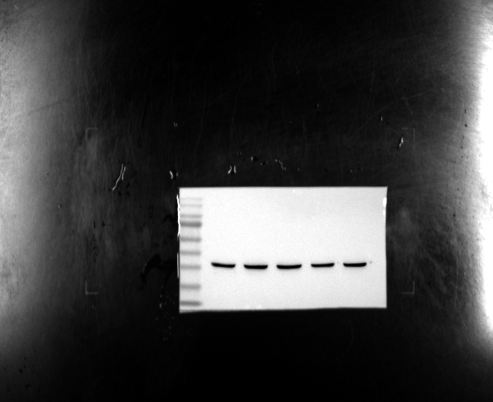


COL2A1.tif GAPDH.tif

Figure 2B


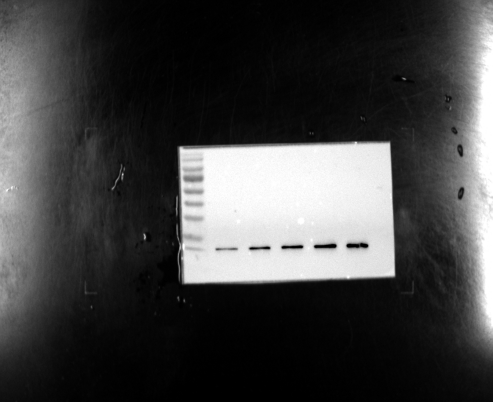

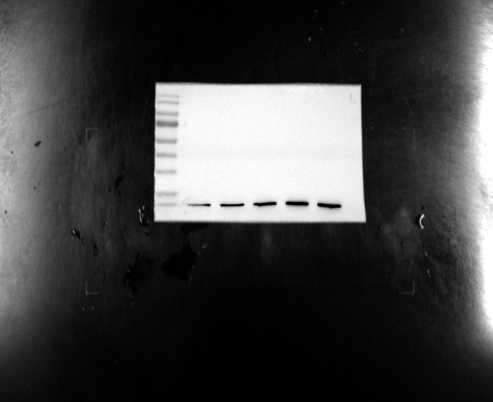


AGR.tif BMP2.tif


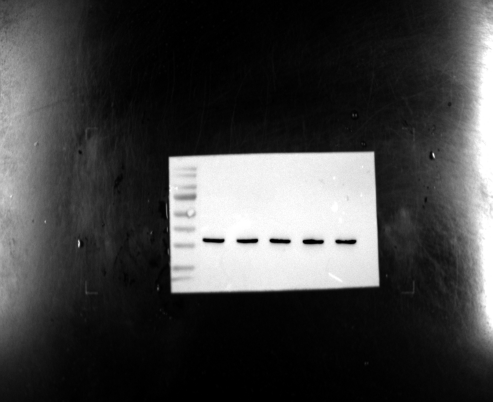

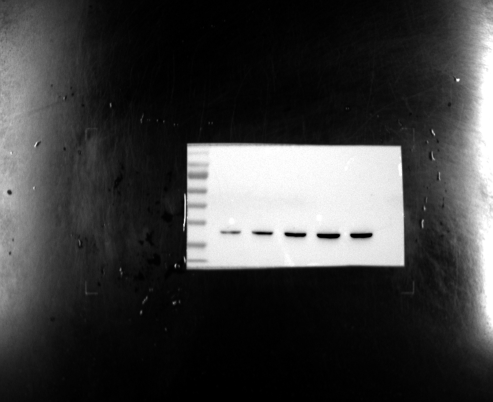


COL2A1.tif GAPDH.tif

Figure 2D


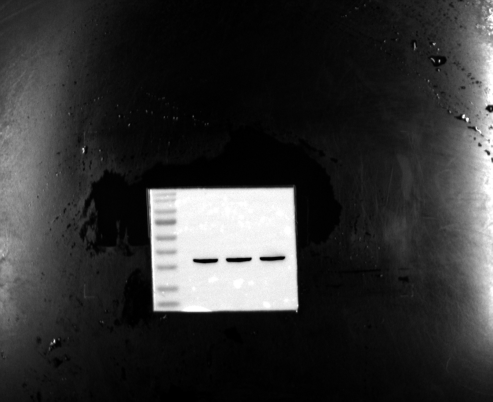

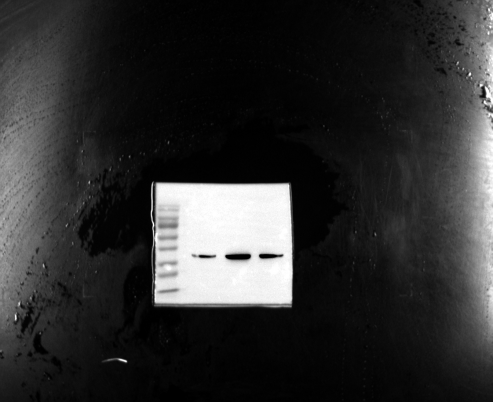


AGR.tif BMP2.tif


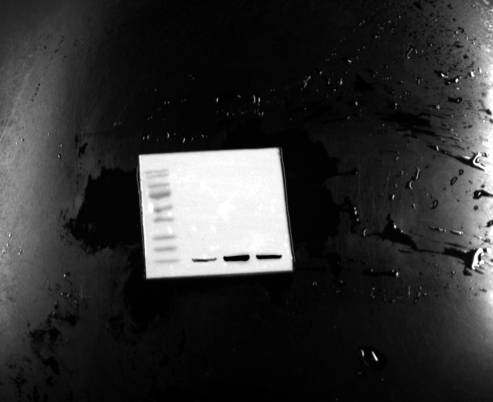

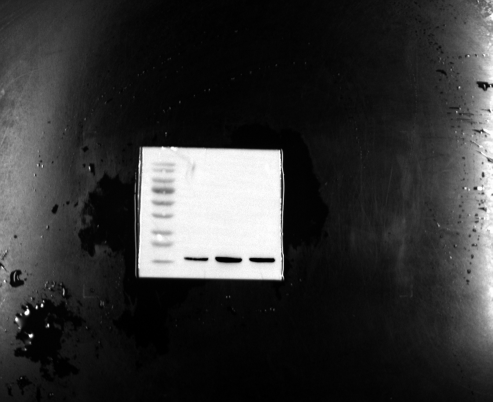


COL2A1.tif GAPDH.tif

Figure 4A


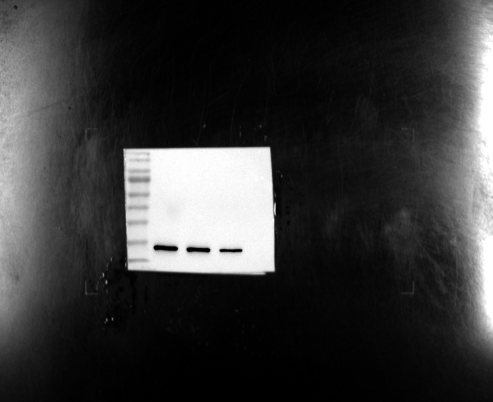

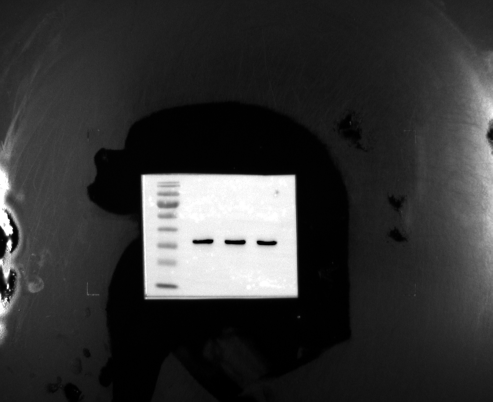


GAPDH.tif GREM1.tif

Figure 5C


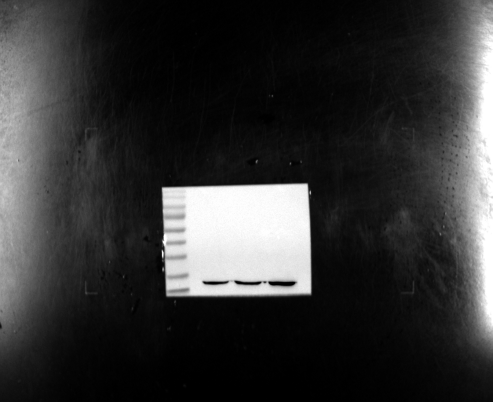

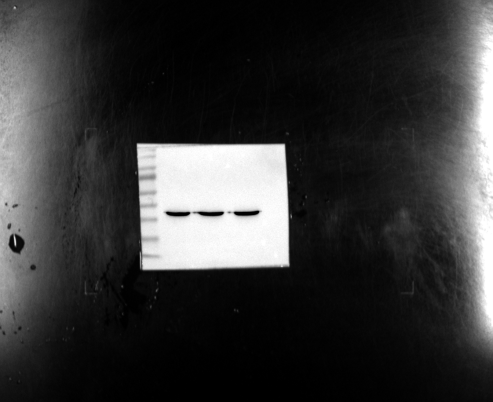


GAPDH.tif GREM1.tif

Figure 5D


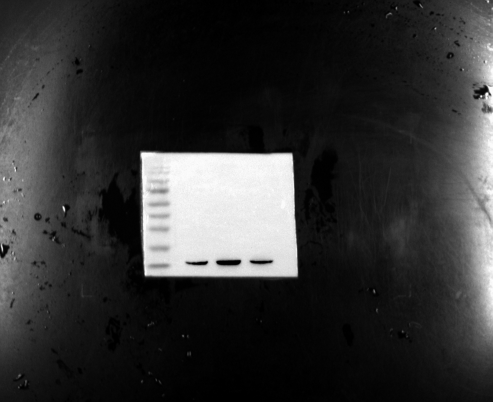

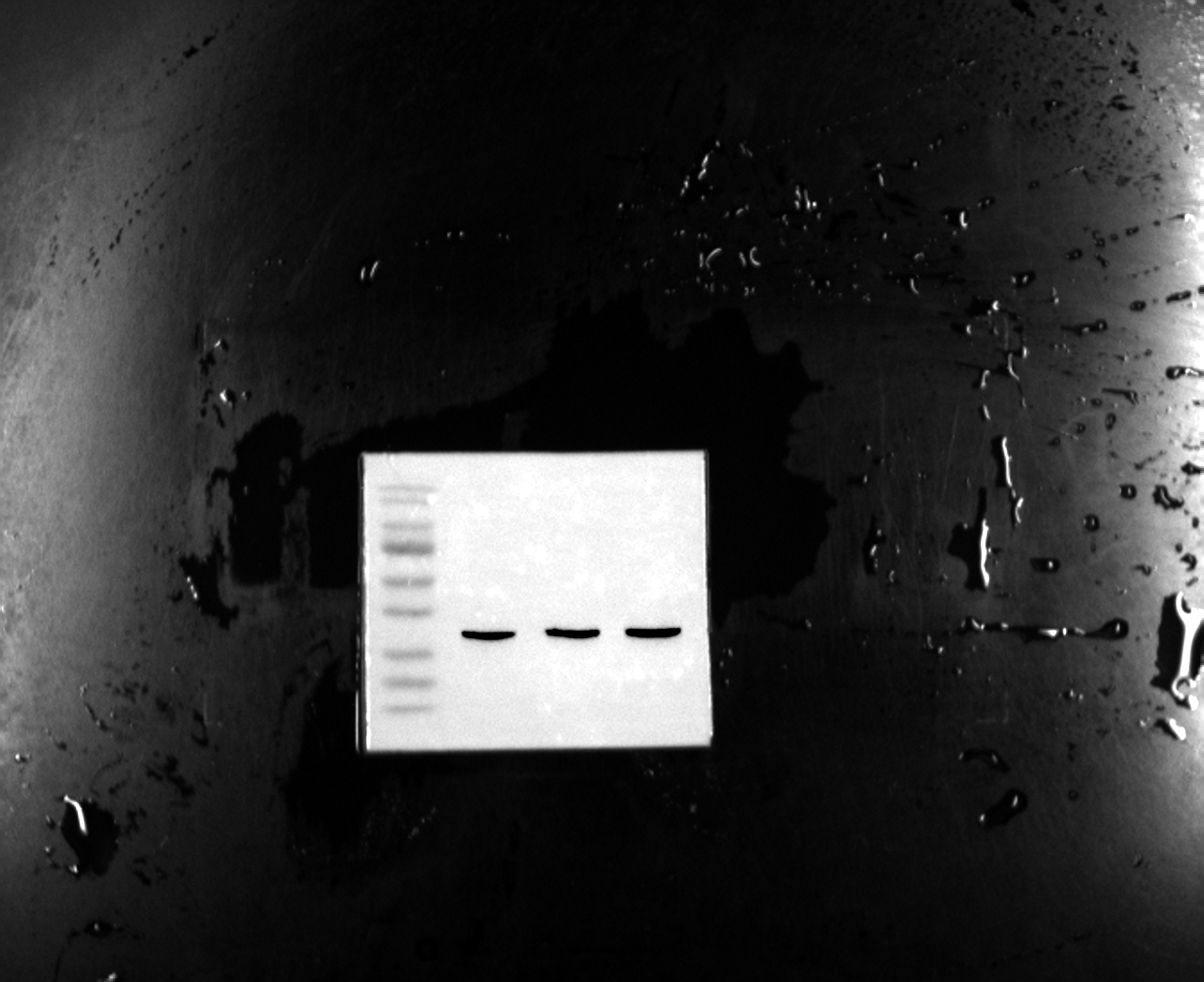


BMP2.tif GAPDH.tif


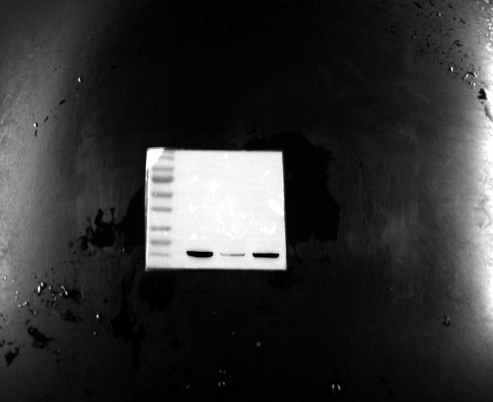


GREM1.tif

Figure 6C


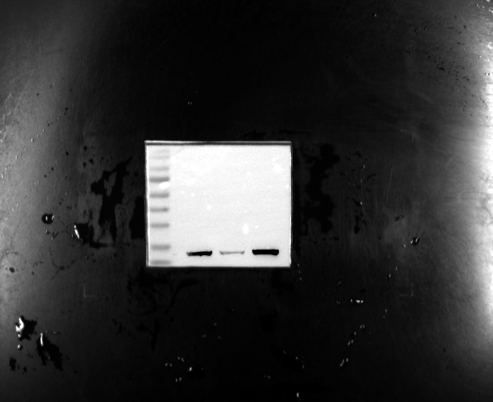

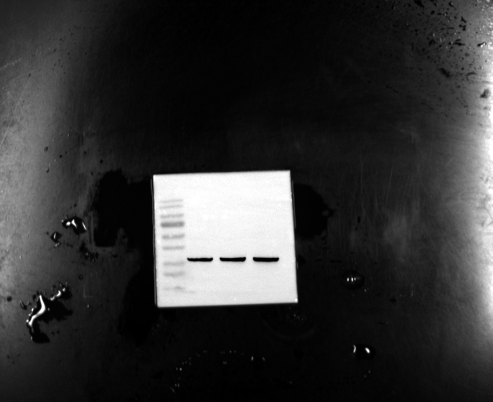


BMP2.tif GAPDH.tif


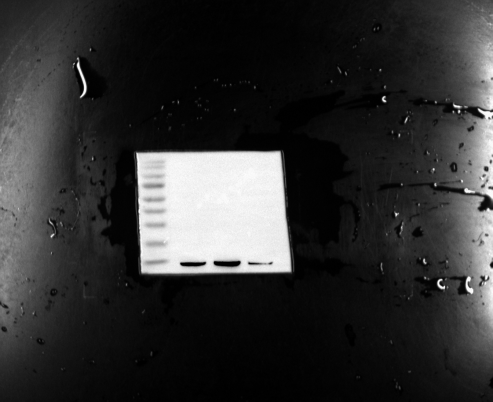


GREM1.tif

Figure 6F
